# A new Bayesian approach incorporating covariate information for heterogeneity and its comparison with HLOD

**DOI:** 10.1186/1471-2156-6-S1-S138

**Published:** 2005-12-30

**Authors:** Swati Biswas, Shili Lin, Donald A Berry

**Affiliations:** 1 Department of Biostatistics, The University of North Texas Health Sciences Center, Fort Worth, TX 76107-2699, USA; 2 Department of Statistics, The Ohio State University, Columbus, OH 43210, USA; 3 Department of Biostatistics and Applied Mathematics, The University of Texas-M. D. Anderson Cancer Center, Houston, TX 77030, USA

## Abstract

We consider a new Bayesian approach for heterogeneity that can take into account categorical covariates, if available. We use the Genetic Analysis Workshop 14 simulated data to first compare the Bayesian approach with the heterogeneity LOD, when no covariate information is used. We find that the former is more powerful, while the two approaches have comparable false-positive rates. We then include informative covariates in the Bayesian approach and find that it tends to give more precise interval estimates of the disease gene location than when covariates are not included. We had knowledge of the simulation models at the time we performed the analyses.

## Background

One of the major difficulties in mapping genes that influence complex traits is locus heterogeneity. A widely used statistic for dealing with locus heterogeneity in linkage analysis is the Heterogeneity LOD (HLOD) score [1]. Recently, Biswas and Lin [2] have proposed a Bayesian approach that accounts for variable levels of heterogeneity across different families by letting each family have its own heterogeneity parameter. This parameter denotes the probability that the family is of the linked type. It was shown through simulations that this approach is more powerful than the HLOD while the two approaches have comparable false-positive rates.

In the current study, we first used the simulated data of the AI population to compare the power and false-positive rates between the Bayesian approach and the HLOD. Then we investigated the performance of a new extension of the Bayesian approach that incorporates informative categorical covariates. We did this by applying the approach to the all-population-combined data using the population indicator as a covariate.

## Methods

### Bayesian approach

This approach is described and investigated in detail by Biswas and Lin [2]. Briefly, suppose there are *k *families in the sample. Let *α*_*j *_be the probability that the *j*^th ^family is of the linked type, *j *= 1, ..., *k*, and let *d *be the position of the disease gene on the chromosome. Here ***α ***= (*α*_1_, ..., *α*_*k*_) is a set of nuisance parameters while *d *is the main parameter of interest. The likelihood of (***α***, *d*) is expressed in terms of mixture distributions, similar to that described in [1]. Suppose there are *N *distances (locations) on the chromosome, labelled as 1, ..., *N*, at which the LOD scores are calculated. Let *I*_*d *_denote the index of distance *d*. Then *I*_*d *_∈ {1, ...., *N*}. The prior distribution for *d *consists of two components: *π*_*d *< ∞ _and *π*_*d *= ∞ _for linkage and no linkage, respectively, on the chromosome of interest. Further, for *d *< ∞, there is a probability distribution of *d *at the *N *distances denoted by *π*_*d *_(*I*_*d*_). Let the prior distribution of *α*_*j *_(_j _= 1, ..., *k*) be *π*_*j*_(*α*_*j*_).

The goal is to obtain the posterior distributions of *α*_*j *_values and *d*. The values of *d *< ∞ (linked subspace) and *d *= ∞ (unlinked subspace) lead to two different models with a different number of parameters. The linked subspace (L) consists of *k*+1 parameters (***α***, *d*) while the unlinked subspace (U) has no parameters, since *d *= ∞ renders the *α *parameters meaningless. To allow moves between these two subspaces, we use the reversible jump Markov chain Monte Carlo sampler [3].

The Markov chain is run initially for a burn-in period of *B *iterations and then for another *T *iterations for estimating the posterior distributions. From the estimated posterior distribution of *d*, we obtain the posterior probability of linkage, , which is the proportion of times the chain is in the L subspace. If  is greater than a certain threshold, *p*_*o*_, we take it as a signal for linkage. In that case, an estimator of the location of the disease gene *under linkage *is the mean, , of the estimated posterior distribution of *d *when the chain is in the L subspace. An interval estimate is obtained by computing a Bayesian credible set (CS) for *d *under linkage.

We use the following prior distributions: *π*_*j*_(*α*_*j*_) is *U*(0,1), *j *= 1, ...,*k*, *π*_*d*< ∞ _= 1/10 (the probability that a randomly chosen chromosome of the simulated data will harbor the disease gene), *π*_*d *= ∞ _= 9/10, and *π*_*d *_(*I*_*d*_) = 1/*N*, *I*_*d *_= 1, ..., *N*. We let *B *= 10,000, *T *= 300,000, following Biswas and Lin [2]. In their study, the values of *π*_*d*< ∞ _and threshold, *p*_*o*_, were, respectively, 1/22 and 0.5 (Bayes rule for the 0–1 loss function), which in turn corresponded to a Bayes factor of 21. Because we are using a different *π*_*d*< ∞_, the Bayes factor of 21 is obtained when *p*_*o *_= 0.7 and so we use this threshold in our analyses.

### Incorporating categorical covariates

One way of incorporating categorical covariates is using a hierarchical model in which the *α *parameters have prespecified hyper-priors. We describe one implementation of this idea as follows: suppose there are *G *groups of families in the sample corresponding to *G *categories of covariate(s). These *G *categories may be *G *levels of one covariate or *G *combinations of levels of two or more covariates. Suppose the *i*^th ^group has *n*_*i*_ families, *i *= 1, ..., *G*. Denote the *α *parameter of the *j*^th^ family in the *i*^th ^group by *α*_*ij*_, *j *= 1, ..., *n*_*i*_, *i *= 1, ..., *G*. Assume that the prior distribution of *α*_*ij*_, *π*_*ij*_(*α*_*ij*_), is *Beta*(*a*_*i *_+ 1,  - *a*_*i *_+ 1) with hyper-parameter *a*_*i *_following *Binomial*(, *φ_i_*). Here, *t*_*i*_, for 0 <*t*_*i *_≤ 1, is a tuning parameter needed only for computational purposes (explained below). The value of *φ*_*i *_is prespecified according to the covariate value in the *i*^th ^category. If the *i*th group is likely to have more linked families, it is assigned a higher value of *φ*_*i*_. The *t*_*i *_values may be set to 1. However, large *n*_*i*_ values lead to large parameters for *Beta *distributions, which make them highly concentrated in a narrow range. This can dramatically increase the computation time for updating the *α*_*ij *_values for some families when using rejection sampling. We use *t*_*i *_= 0.1 for all *i*.

## Results

We used the following genetic model, where D denotes the disease allele: P(D) = 0.2, and penetrances for genotypes dd, Dd, and DD are 0.05, 0.5, and 0.7, respectively. This is a kind of incomplete penetrance model that one might use as an approximation to the true but unknown complex model. The data used are microsatellite markers and affection statuses. We used GENEHUNTER version 2.1_r5 beta [4] to compute multipoint LOD and HLOD scores at every 1 cM, starting from 10 cM above the first marker and ending at 10 cM beyond the last marker on a chromosome. We let the GENEHUNTER (with its max bits option set to 18) drop any members automatically in its computations.

### Comparison of Bayesian approach with HLOD

We analyzed all 100 replicates and all ten chromosomes for population AI. The number of replicates that give a linkage signal at any location on each chromosome using the Bayesian approach and HLOD are shown in Table 1. For HLOD, a cut-off of 3.7 is used to declare linkage, following the recommendation of Greenberg and Abreu [5] for multipoint analysis. According to the simulation model, there are four disease loci, one each on chromosomes 1, 3, 5, and 9, while there are two modifying loci, one each on chromosomes 2 and 10. Table 1 shows that the Bayesian approach is more powerful than the HLOD. The Bayesian approach (with a threshold of 0.7) gives one false positive while the HLOD gives zero false positives. Although this false-positive rate for the Bayesian approach is very low (1/400 = 0.0025) in the absolute sense, we also explored increasing its cut-off, *p*_*o*_, to 0.73 (results also shown in Table 1) so that its false-positive rate becomes zero; we see that the Bayesian approach retains higher powers than the HLOD. We also note that all 95% CSs for the Bayesian approach (results not shown) contain their corresponding disease gene locations when linkage is detected on chromosomes 1, 5, 9, and 10. This result cannot be fully asserted for chromosome 3 because the "answers" provided for the simulation model only indicated that the disease gene on this chromosome lies beyond the last SNP marker, and hence its exact location is unknown to us.

### Analysis with covariate

We used the population indicator (which takes the values AI, KA, DA, or NYC) as a covariate. These populations differ in the diagnostic schemes used, which are based on three broad categories of symptoms: communally-shared emotions (COM), behavioral (BEH), and anxiety-related (ANX). In AI and NYC, a person is considered to be affected by Kofendrerd Personality Disorder if he/she has any of the three types of symptoms. In KA, the criterion is the presence of COM or ANX, while in DA only BEH matters. NYC consists of extended pedigrees in contrast to the other three populations that comprise nuclear families only. Furthermore, NYC differs from the others in its ascertainment scheme. The varying diagnostic and ascertainment schemes can be expected to give rise to some heterogeneity across populations. Specifically, if there is a locus that influences COM and/or ANX but not BEH, then DA is not expected to contain information about this locus while KA is the most homogeneous and hence most informative for detecting the locus. On the other hand, DA is most informative for a locus influencing BEH. Also, although both AI and NYC are phenotypically heterogeneous, NYC families may contain more linkage information because the families ascertained are extended pedigrees with at least four affected members.

In our first analysis, we found linkage signals on chromosomes 1, 3, 5, and 9 in numerous replicates. Although we used population AI only, all three types of symptoms (and hence all loci influencing them) are present in the AI families, thus we decided to focus on these chromosomes only. Following the above reasoning concerning the informativeness of populations for different types of loci, we considered two possible sets of binomial parameters (*φ_i_*) for (AI, KA, DA, NYC): 1) (0.5, 0.4, 0.9, 0.6) and 2) (0.5, 0.9, 0.2, 0.6). We analyzed the first 25 replicates with both sets of covariate settings and without covariate. The results for the first 5 replicates only are shown in Table 2; those for the other 20 replicates are similar and thus are omitted from the table. For the results with covariate, we have listed the results for the set of *φ_i _*parameters that gave a higher posterior probability of linkage (set 1 for chromosomes 1 and 3; set 2 for chromosomes 5 and 9).

From Table 2, we see that for most of the chromosome 1, 5, and 9 replicates, including covariate information leads to narrower interval estimates (CSs). This observation also holds true for all 25 replicates that we analyzed. More specifically, with covariate information, the proportions that have narrower CSs are 0.8, 0.5, and 0.6 for chromosomes 1, 5, and 9, respectively, whereas without covariate information, the corresponding proportions are only 0.12, 0.23, and 0.2. However, for chromosome 3, it appears that including the covariate is not useful and may even lead to some loss of power (see below).

## Conclusion

Our study shows that the Bayesian approach is more powerful than the HLOD while the two have comparable false-positive rates, consistent with Biswas and Lin [2]. From Table 1, we observe that the relative power gains for detecting the disease loci using the Bayesian approach (with a cut-off of 0.73) compared to the HLOD range from 35% to 100%, a finding also similar to that in Biswas and Lin [2]. This is noteworthy because, unlike their simulation models, all the underlying disease models of the GAW14 data are epistatic and do not follow a heterogeneity model. This shows that our Bayesian approach performs better than the HLOD under more complicated disease models also. We note that there is another Bayesian approach for mapping under heterogeneity [6], although it differs from the approach we propose in many significant aspects, including its basic formulation.

According to the simulation model, the disease gene on chromosome 1 (D1) influences BEH strongly and either ANX or COM moderately, with no effect on the other type of symptom. The genes on chromosomes 5 (D3) and 9 (D4) influence COM and ANX only while the one on chromosome 3 (D2) affects all symptoms, with a slightly stronger effect on BEH. Our results are consistent with this simulation model, since of the two sets of *φ_i _*values, we expect, the first set to target D1 and the second set to point toward D3 and D4. This illustrates how prior information (in this case the diagnostic and ascertainment schemes) can be used to form informative covariates that refine the linkage analysis by yielding narrower CSs and even uncovering signals for linkage that might otherwise be missed. However, we note that even when no covariate is included, the linkage signals in most of the replicates with all populations combined are so strong (indicated by  ≈ 1 in Table 2) that little room is left for further improvement when a covariate is included. This is the most likely reason why including the covariate led to only minor reductions in the widths of CSs (mostly 1 cM). Nevertheless, since this reduction is consistently seen across the majority of the 25 samples analyzed, it appears that it is truly due to the effect of the covariate rather than random variation. In any case, further evaluations and refinements are needed to assess the benefits of incorporating covariates. For instance, a more objective method for choosing the *φ_i _*values, such as through the use of an empirical Bayes approach, should be explored. Such an approach may also reveal when a covariate is not informative, as it was for the chromosome 3 data. In this case the inclusion of the covariate should not be recommended, because otherwise it may lead to wider CSs and even a loss of power (as in the case of replicate 2 of chromosome 3 in Table 2).

The approximate locations of D1, D2, D3, and D4 are, respectively, 169, 299 (location of the last SNP), 5, and 6 cM on their respective chromosomes based on the microsatellite map. Compared with the CSs in Table 2, we see that all of them are able to capture their corresponding true disease gene locations. We conclude that the Bayesian approach is a powerful tool for localizing a disease gene to a narrow interval.

## Abbreviations

ANX: Anxiety-related

BEH: Behavioral

COM: communally-shared emotions

CS: Credible set

HLOD: Heterogeneity LOD

## Authors' contributions

SB and SL developed the methodology. SB carried out the applications and drafted the manuscript. DAB provided insightful comments for the presentation of results. SL and DAB were involved in the critical revision of the manuscript. All authors read and approved the final manuscript.

## Figures and Tables

**Table 1:** Numbers of replicates (out of 100 for each chromosome) that give linkage signals for population AI.

	Chromosome									
	
	1^a^	2^b^	3^a^	4	5^a^	6	7	8	9^a^	10^b^
Bayesian ( > 0.7)	30	0	31	0	21	0	0	1	10	1
Bayesian ( > 0.73)	27	0	31	0	19	0	0	0	10	1
HLOD (HLOD > 3.7)	20	0	23	0	10	0	0	0	5	0

^a^Chromosome harbors the disease gene.

^b^Chromosome harbors the modifying loci.

**Table 2:** Bayesian approach results^a ^with (Y) or without (N) "population" covariate.

Replicate Number	Covariate	Chromosome 1			Chromosome 3		
			
				95% CS			95% CS
1	Y	1.000	166.70	(163, 171)	1.000	297.99	(294, 304)
	N	1.000	166.68	(163, 172)	1.000	297.32	(294, 302)

2	Y	0.983	172.43	(165, 178)	0.696	-	-
	N	0.947	171.41	(161, 178)	0.787	299.07	(291, 306)

3	Y	1.000	169.67	(165, 175)	0.993	298.92	(292, 306)
	N	1.000	169.86	(165, 176)	0.994	297.60	(292, 306)

4	Y	1.000	164.30	(161, 169)	0.999	300.12	(294, 306)
	N	1.000	164.22	(159, 170)	0.999	298.90	(293, 306)

5	Y	1.000	169.21	(166, 173)	1.000	296.91	(294, 301)
	N	1.000	168.71	(165, 173)	1.000	296.82	(294, 300)

		Chromosome 5			Chromosome 9		
			

				95% CS			95% CS

1	Y	0.996	0.00	(0, 5)	0.998	1.76	(0, 10)
	N	0.971	0.00	(0, 6)	0.981	2.22	(0, 12)

2	Y	1.000	8.02	(4, 12)	1.000	6.64	(0, 11)
	N	1.000	8.68	(4, 13)	0.998	6.61	(0, 13)

3	Y	1.000	3.14	(0, 12)	0.735	0.00	(0, 10)
	N	1.000	3.85	(0, 12)	0.172	-	-

4	Y	0.108	-	-	0.973	0.00	(0, 9)
	N	0.297	-	-	0.995	1.43	(0, 9)

5	Y	0.998	4.02	(0, 12)	0.001	-	-
	N	0.998	5.33	(0, 12)	0.001	-	-

^a^ and 95% CS are the mean and (2.5^th^, 97.5^th^) percentiles of the estimated posterior distribution of *d under linkage*, respectively.  and CS are reported only if posterior probability of linkage  > 0.7. Note that the 0 in the lower limits of the CS and the  values are due to the truncations of negative map positions that resulted from consideration of positions 10 cM before the first marker, for computational reasons.
